# Evaluation of submaximal endurance in young children living with HIV

**DOI:** 10.4102/sajp.v78i1.1613

**Published:** 2022-02-21

**Authors:** Joanne Potterton, Renate Strehlau, Stephanie Shiau, Nicolette Comley-White, Louise Kuhn, Michael Yin, Stephen Arpadi

**Affiliations:** 1 Department of Physiotherapy, Faculty of Health Sciences, University of the Witwatersrand, Johannesburg, South Africa; 2 Empilweni Services and Research Unit, Rahima Moosa Mother and Child Hospital, Department of Paediatrics and Child Health, School of Clinical Medicine, Faculty of Health Sciences, University of the Witwatersrand, Johannesburg, South Africa; 3 Department of Biostatistics and Epidemiology, Rutgers University, Piscataway, NJ, United States of America; 4 Gertrude H. Sergievsky Centre, Columbia University Irving Medical Center, New York, United States of America; 5 Department of Infectious Diseases, Columbia University Irving Medical Center, New York, United States of America

**Keywords:** HIV, children, endurance, physical activity, paediatric

## Abstract

**Background:**

There is growing concern about the long-term *sequelae* [a condition which is the consequence of a previous disease or injury] of perinatally acquired human immunodeficiency virus (HIV). Children living with HIV (CLHIV) present with cardiopulmonary impairments and decreased physical activity which may be due to poor endurance.

**Objectives:**

Our study aimed to investigate the sub-maximal endurance of CLHIV compared to a non-infected comparison group.

**Methods:**

In this cross-sectional descriptive study 346 CLHIV, between ages five and eleven years, were assessed using the Six Minute Walk Test (6MWT). Blood pressure, heart rate and oxygen saturation were measured pre-test, immediately post-test and five minutes post-test. Clinical and anthropometric data were recorded. Height and weight were assessed using a stadiometer and a digital scale, respectively.

**Results:**

175 CLHIV (52% female) and 171 children without HIV (46% female) participated. All children were Black African. The CLHIV all initiated antiretroviral therapy (ART) at a young age (mean 8.7 months, standard deviation 6.7) and their disease was well controlled (viral load < 1000copies/ml). There were no statistically significant differences in submaximal endurance between the two groups (*p* = 0.831). Age of starting ART and stunted growth were negatively associated (*r* = -2.8 (*p* = 0.019) and *r* = -46.1 (*p* = 0.027), respectively) with distance walked in the 6MWT by girls living with HIV.

**Conclusion:**

CLHIV who initiate ART early with well-controlled disease are able to attain submaximal endurance levels similar to their uninfected peers.

**Clinical implications:**

Endurance and physical activity should be monitored in CLHIV. Submaximal endurance levels may improve with age and biological maturation.

## Introduction

Paediatric human immunodeficiency virus (HIV) remains a challenge globally, especially in sub-Saharan Africa which is home to over 90% of children living with HIV (CLHIV) (UNAIDS 2020). Early initiation of antiretroviral therapy (ART) has been shown to significantly reduce mortality as well as morbidity and children perinatally infected with HIV are living longer, healthier lives than in the pre-ART era (Lowenthal et al. 2014). There has been a necessary shift from acute care in infants and children to managing the long-term health of older children and adolescents living with HIV. The long-term, multisystem impact of chronic inflammation because of HIV is becoming ever more evident and may be associated with the development of new complications such as cognitive (Natukunda et al. 2020) and metabolic abnormalities (Barlow-Mosha et al. 2013), as well as increased risk of cardiopulmonary disease (Githinji et al. 2019). There is growing concern over the long-term physical sequelae of living with HIV (Laughton et al. 2013).

Children living with HIV are often stunted, and wasted, and poor growth has been associated with decreased physical functioning (Brassell & Potterton 2019; Wong et al. 2016). Stunted growth can continue through childhood into adolescence (Jesson et al. 2019). Poor growth mediates a delay in sexual maturation and thus onset of puberty (Iloh et al. 2017). Decreased bone mineral density and deficits in bone architecture have been described in CLHIV (Arpadi et al. 2016; Shiau et al. 2020).

There is increasing evidence that children and adolescents living with HIV have a high prevalence of lung function impairment, despite being on ART, which presents predominantly as irreversible lower airway obstruction and reduced aerobic function (Githinji et al. 2017; Githinji, Gray & Zar 2018; Rylance et al. 2016). The CLHIV who were perinatally infected have also shown reduced cardiac function that may result from direct viral effects or as a consequence of long-term ART exposure. These cardiac impairments, which may be sub-clinical, are a growing concern as CLHIV are exposed to ART for extended periods (Githinji et al. 2019; Lipshultz et al. 2013; Sims Sanyahumbi et al. 2017). Cardiopulmonary impairments, along with potential renal dysfunction and decreased bone mineral density (Shiau et al. 2020), could impact negatively on participation in physical activity. Levels of moderate to vigorous physical activity (MVPA) have been noted to be decreased in CLHIV (De Lima et al. 2017, 2019; Wong et al. 2016), with the activity levels of girls being a particular concern (Wong et al. 2016). Poor endurance levels may be a factor contributing to this decrease in MVPA, but this relationship needs to be investigated further.

The 6-minute walk test (6MWT) is a commonly used measure of submaximal endurance, which is said to be a good reflection of the energy demands of everyday activities of daily living. It has been used successfully in a number of paediatric populations, including young CLHIV (Sims Sanyahumbi et al. 2017; Walker 2015), and is a popular tool in clinical settings because of its ease of administration and cost-effectiveness (‘ATS Statement’ 2002; Cacau et al. 2016).

The aim of our study was to determine the submaximal endurance levels of CLHIV who initiated ART at an early age. Secondary objectives of the study were to determine the anthropometric status and sexual maturity of the children, and to determine whether these were associated with submaximal endurance levels.

## Methods

The CHANGES Bone Study followed 219 HIV-infected children and 219 HIV-uninfected children in Johannesburg, South Africa (Arpadi et al. 2016). At a cross-sectional visit, children aged 5–11 years and their caregivers were invited to participate in our sub-study. All children in the CHANGES study were eligible to participate (inclusion criteria for the CHANGES study have been previously published [Arpadi et al. 2016]). The study was explained and an information sheet was provided. Caregivers provided signed consent and children aged 7 years and above who agreed to participate signed assent.

A sample of 175 CLHIV and 171 children without HIV agreed to participate, and were assessed for submaximal endurance by one of the two physiotherapists involved in our study. Children were not assessed if they had a raised temperature > 37°C, respiratory rate > 24 breaths per min or oxygen saturation level < 90% on the day of testing.

Endurance was assessed using the 6MWT. The American Thoracic Society guidelines for administering the test were adhered to (‘ATS Statement’ 2002). Because of lack of space, a 10m oval track was used instead of a 30m walkway, and the distance walked by each child was recorded in meters. Each child’s heart rate was recorded using a heart rate monitor (Polar H10), which was attached around the child’s chest throughout the test. A pre-test reading was taken to record resting heart rate (beats per minute). Average and maximum heart rate for the test was recorded. A post-test reading was taken immediately and 5 min after completing the walk test. Systolic and diastolic blood pressure (BP) were measured using a paediatric cuff and sphygmomanometer placed on the child’s left arm and were recorded pre-test and post-test. Percent oxygen saturation was also measured pre-test and post-test, using a pulse oximeter (DIGIT^®^) placed on the child’s index finger.

Clinical and anthropometric data, including height (cm) and weight (kg), were extracted from the child’s clinical file. Z scores were calculated using World Health Organization (WHO) norms, and stages of sexual maturation were assessed using Tanner staging (Marshall & Tanner 1969, 1970). Underweight was defined as weight-for-age Z (WAZ) < –2, and stunted was defined as height-for-age (HAZ) < –2. We compared measurements of CLHIV to a comparison group without HIV, which included uninfected siblings, household members or children attending the clinic from a similar socioeconomic background (Arpadi et al. 2016).

Descriptive statistics were used to summarise and report the data from the two groups. A linear regression univariate correlation analysis was performed to investigate associations between anthropometric and clinical data and the 6MWT. Sex-stratified analyses were reported. The results were reported as coefficient estimates (*p*-value; two tailed) with significance set at *p* ≤ 0.05.

### Ethical considerations

Ethical approval to conduct the study was granted by the Human Research Ethics Committee (Medical) of the University of the Witwatersrand. Reference number: M120871.

## Results

The results for 175 CLHIV and 171 children not infected with HIV were analysed. The distribution of age and sex did not differ between the two groups. The anthropometric data stratified for sex is presented in Table 1.

**TABLE 1:** Age and anthropometric measures stratified by sex.

Characteristic or measurement	Boys								*p*	Girls								*p*
	Living with HIV (*N* = 84)				Uninfected (*N* = 93)					Living with HIV (*N* = 91)				Uninfected (*N* = 78)				
	*n*	Mean	%	s.d.	*n*	Mean	%	s.d.		*n*	Mean	%	s.d.	*n*	Mean	%	s.d.	
Age in years	-	9.4	-	2.0	-	9.3	-	1.9	0.7465	-	9.1	-	1.8	-	9.1	-	2.0	0.983
**Anthropometric measurement values closest to day of assessment of endurance**																		
WAZ	-	−0.93	-	0.87	-	−0.25	-	1.05	< 0.001*	-	−0.68	-	1.03	-	0.01	-	1.23	0.001*
Underweight	6	-	10.5	-	2	-	3.3	-	0.156	9	-	13.2	-	1	-	1.8	-	0.021*
HAZ	-	−1.13	-	0.92	-	−0.62	-	0.91	< 0.001*	-	−0.90	-	1.76	-	−0.43	-	0.88	0.027*
Stunted	13	-	15.5	-	5	-	5.4	-	0.044*	16	-	17.6	-	2	-	2.6	-	0.002*
BMI (kg/m^2^)	-	16.0	-	2.0	-	16.5	-	2.6	0.096	-	16.1	-	2.5	-	17.0	-	3.4	0.071
BAZ	-	−0.38	-	0.95	-	−0.06	-	1.19	0.015*	-	−0.28	-	1.37	-	0.05	-	1.31	0.049*
Tanner stage 1	66	-	78.6	-	78	-	83.9	-	0.441	69	-	75.8	-	56	-	71.8	-	0.600

WAZ, weight-for-age *z*-score; HAZ, height-for-age *z*-score; BMI, body mass index; BAZ, BMI-for-age *z*-score; HIV, human immunodeficiency virus; s.d., standard deviation.

* , statistical significance set at *p* ≤ 0.05.

The WAZ and HAZ scores were significantly lower in both males and females living with HIV compared to males and females without HIV. A higher proportion of girls with HIV had wasting (WAZ more than 2 standard deviation [s.d.] below mean on WHO growth charts) than girls without HIV. Stunting (HAZ more than 2 s.d. below mean on WHO growth charts) and lower BAZ scores were both significantly more prevalent in boys and girls living with HIV compared to their uninfected peers.

Table 2 presents the laboratory test results for the CLHIV. These results were those closest to the date of endurance assessment.

**TABLE 2:** Laboratory test results for children living with human immunodeficiency virus closest to the endurance assessment visit.

Laboratory tests	Boys living with HIV *N* = 84							Girls living with HIV *N* = 91							*p*
	*N*	Mean	Range	Median	%	s.d.	IQR	*N*	Mean	Range	Median	%	s.d.	IQR	
**Viral load (copies/ml)**	-	-	-	-	-	-	-	-	-	-	-	-	-	-	0.691
TND or LDL	65	-	-	-	77.4	-	-	68	-	-	-	74.7	-	-	
21–1000	16	-	-	-	19.1	-	-	17	-	-	-	18.7	-	-	
> 1000	3	-	-	-	3.6	-	-	6	-	-	-	6.6	-	-	
CD4 count (cells/*mm*^3^)	-	-	16.5–1624.0	-	-	-	-	-	-	169.0–2358.0	-	-	-	-	NA
CD4 count (cells/*mm*^3^)	-	983.9	-	-	-	313.0	-	-	1070.6	-	-	-	384.0	-	0.105
CD4 count (cells/*mm*^3^)	-	-	-	960.5	-	-	752.0, 1237.5	-	-	-	1032.0	-	-	812.0, 1280.0	0.191
**CD4 count (cells/mm^3^)**	-	-	-	-	-	-	-	-	-	-	-	-	-	-	0.362
< 750	21	-	-	-	25.0	-	-	18	-	-	-	19.8	-	-	
750–1000	25	-	-	-	29.8	-	-	22	-	-	-	24.2	-	-	
≥ 1000	38	-	-	-	45.2	-	-	51	-	-	-	56.0	-	-	
CD4 (%)	-	-	17.66–49.22	-	-	-	-	-	-	9.81–56.59	-	-	-	-	NA
CD4 (%)	-	36.39	-	-	-	7.29	-	-	38.39	-	-	-	7.33	-	0.072
CD4 (%)	-	-	-	37.06	-	-	31.17, 42.13	-	-	-	38.08	-	-	34.10, 43.69	0.102
**CD4 (%)**	-	-	-	-	-	-	-	-	-	-	-	-	-	-	0.027
< 25	5	-	-	-	6.0	-	-	5	-	-	-	5.5	-	-	
25–30	15	-	-	-	17.9	-	-	4	-	-	-	4.4	-	-	
30–35	15	-	-	-	17.9	-	-	15	-	-	-	16.5	-	-	
≥ 35	49	-	-	-	58.3	-	-	67	-	-	-	73.6	-	-	
Age at ART start in months	-	9.0	-	-	-	6.7	-	-	8.3	-	-	-	6.8	-	0.485

IQR, inter-quartile range; HIV, human immunodeficiency virus; s.d., standard deviation; ART, antiretroviral therapy; TND, target not detected; LDL, lower than detectable limit; NA, not applicable.

The majority of children were virally suppressed (i.e. < 1000 copies/mL) and were not immune deficient based on CD4 counts. The mean age for ART initiation for boys and girls combined was 8.7 months (s.d. 6.7). There were significantly more girls with CD4% over 35%, although there was not a significant difference in the mean CD4% between boys and girls

The results of the 6MWT, stratified for sex, are presented in Table 3.

**TABLE 3:** Blood pressure, heart rate, oxygen saturation and 6-minute walk test results for children living with human immunodeficiency virus and the uninfected comparison group.

Measurement	Boys				*p*	Girls				*p*
	Living with HIV *N* = 84		Uninfected *N* = 93			Living with HIV *N* = 91		Uninfected *N* = 78		
	Mean	s.d.	Mean	s.d.		Mean	s.d.	Mean	s.d.	
Total distance walked (m)	418.9	97.7	408.8	95.1	0.488	371.3	77.4	386.6	86.4	0.228
Pre-test heart rate (beats/min)	82.7	13.4	76.5	11.4	0.001*	84.5	11.9	84.1	13.4	0.822
Immediate post-test heart rate (beats/min)	89.1	13.6	82.3	11.3	< 0.001*	91.0	13.9	91.6	13.5	0.776
5-min post-test heart rate (beats/min)	85.7	12.9	79.3	11.8	< 0.001*	87.9	12.8	87.6	12.7	0.887
Average heart rate (beats/min)	123.8	15.3	116.6	12.6	< 0.001*	121.6	15.2	122.7	15.1	0.651
Maximum heart rate (beats/min)	144.0	26.6	133.2	21.3	0.003*	136.3	23.7	139.8	22.0	0.349
Pre-test Sats (%)	96.8	3.3	97.3	1.6	0.156	97.1	2.7	96.8	2.8	0.546
Immediate post-test Sats (%)	97.5	1.8	97.3	2.0	0.617	97.2	2.9	97.3	1.7	0.968
5-min post-test Sats (%)	97.3	1.9	97.3	1.3	0.951	97.4	1.6	97.3	1.4	0.624

Sats, oxygen saturation; HIV, human immunodeficiency virus; s.d., standard deviation.

* , statistical significance set at *p* ≤ 0.05.

When looking at results for boys and girls combined, there were no significant differences between the two groups for pre-test and post-test BP or oxygen saturation. The CLHIV had significantly higher pre-test (*p* = 0.007) and post-test (*p* = 0.006) heart rates than the HIV-uninfected group. The distance walked on the 6MWT was similar in both groups (*p* = 0.831), but markedly lower than reported in other studies (Cacau et al. 2016).

Once the results were stratified for sex, it becomes apparent that the differences noted in heart rate apply to boys and not to girls, with boys living with HIV having significantly higher heart rates before and after the 6MWT than the boys without HIV. However, there were no significant differences in 6MWT distance (6MWD) between girls with and without HIV.

For the CLHIV, univariate analysis for anthropometric measurements and laboratory measurements on the 6MWD was conducted. Sex stratified analyses are reported. The results are reported as a coefficient estimate (*p* value). Table 4 presents the results.

**TABLE 4:** Univariate analysis for factors associated with the 6-minute walk test distance.

Variables	All children with HIV *N* = 175		Boys with HIV *N* = 84		Girls with HIV *N* = 91	
	6MWD	*p*	6MWD	*p*	6MWD	*p*
Age of ART initiation (months)	−1.8	0.060	−0.8	0.603	−2.8	0.019*
HAZ	4.8	0.247	9.4	0.332	4.3	0.326
Stunted (ref: no stunted)	−29.8	0.060	−3.7	0.877	−46.1	0.027*
BMI	−0.6	0.820	−5.9	0.239	1.7	0.595
BAZ	0.894	0.857	−8.3	0.362	3.8	0.503
Tanner stage 1 (ref: > 1)	0.4	0.984	2.0	0.948	−7.4	0.777
CD4 count:750–1000 (ref: < 750)	−19.7	0.246	−12.5	0.596	−31.1	0.195
CD4 count: < 1000 (ref: < 750)	−17.0	0.264	−26.1	0.233	−13.7	0.513
Viral load: 21–1000 (ref: LDL)	2.7	0.862	−6.7	0.762	1.2	0.953
Viral load: > 1000 (ref: LDL)	2.9	0.914	−58.1	0.214	47.3	0.137

6MWD, six-minute walk distance; HAZ, height-for-age *z*-score; BMI, body mass index: BAZ, BMI-for-age *z*-score; LDL, lower than detectable limit; HIV, human immunodeficiency virus.

* , statistical significance set at *p* ≤ 0.05.

For boys, there were no associations between any of the demographic, anthropometric and clinical variables and the distance walked on the 6MWT. However, for girls, stunting and starting ART later were associated with a shorter distance walked on the 6MWT suggesting poorer endurance levels in these girls.

## Discussion

The submaximal endurance of young children perinatally infected with HIV who started ART early and whose disease is well controlled is similar to that of HIV-uninfected children from the same socioeconomic background in South Africa. The children in our study who were living with HIV were all initiated on ART within the first 18 months of life, and they were generally in good health, with over 75% of the participants having a non-detectable viral load.

There was no difference between the two groups in 6MWT results in terms of the distance walked. These findings are similar to the findings by Githinji et al. (2017) who in their study investigated the lung function of adolescents aged 9–14 years, perinatally infected with HIV in Cape Town. The total distance walked in their study was slightly higher, but their participants were older with a mean age of 12 (s.d. 1.6) years.

A study conducted in Malawi (Sims Sanyahumbi et al. 2017) found a significant difference in 6MWT results between CLHIV and children without HIV infection between the ages of 4 and 18 years, with the children with HIV walking a shorter distance in 6 min. A notable difference between the children in their study and ours was that the children in their study were much older when ART was initiated, not all children in their study were on ART, and disease was not as well controlled as evidenced by viral load and CD4 count. A further study from rural South Africa found a significant difference between groups with CLHIV walking a significantly shorter distance on the 6MWT than their HIV uninfected peers (Walker 2015). The majority of these children (80%) were relatively well (WHO stage 1) and time on ART varied from 6 to 91 months (Walker 2015).

There were no differences observed between the groups for BP and oxygen saturation. However, resting and post exercise heart rates were significantly higher in the CLHIV, particularly for boys. Githinji et al. (2017) and Walker (2015) found the opposite in their studies with the HIV-infected participants having lower heart rates. The children in these studies were similar in age to those in our study ranging from 9 to 14 years in Githinji et al.’s study and from 7 to 10 years in Walker’s study. The cardiovascular response of CLHIV needs to be investigated further.

The 6MWT is a submaximal endurance test and not sensitive enough to pick up small changes in cardiopulmonary function (Githinji et al. 2017). Other studies that have found differences in endurance between CLHIV and children without HIV have used maximum volume of oxygen consumption (VO2 max) as the outcome measure. Somarriba et al. (2013) found that CLHIV had lower VO2 peak (Somarriba et al. 2013) as did De Lima et al. (2017). De Lima et al. found VO2 max to be even lower in CLHIV who were not on ART. Other studies that looked at chronic lung disease in adolescents with perinatal HIV reported reduced exercise tolerance in 21% of the participants, with 13% being hypoxic at rest and 29% hypoxic on exercise (Ferrand et al. 2012). Additionally, 43% of a group of adolescents with HIV presented with dyspnoea on exertion (Miller et al. 2013). It is noteworthy that in both Ferrand et al.’s and Miller et al.’s studies, the participants had a delayed diagnosis of perinatal HIV and that only 69% and 71%, respectively, were on ART. In our study, the participants had all initiated ART when they were 18 months old and were well managed.

Wong et al. (2016), using the same cohort of children included in our study, found that CLHIV spent less time in vigorous physical activity—especially girls (Wong et al. 2016). Martins et al. (2017) reported that CLHIV between 10 and 15 years of age had lower overall physical activity scores than their healthy peers. Physical education lessons at school provided an opportunity for these children to participate in physical activity (Martins et al. 2017). De Lima et al. (2017) also found that CLHIV between 8 and 15 years of age spent less time in 5 min and 10 min bouts of MVPA, although total MVPA was similar to the control group. The fact that less time is spent in MVPA may indicate decreased endurance as evidenced by the low VO2 max in this group (De Lima et al. 2017). The growth and activity levels of girls with HIV warrant particular attention as evidenced by the decreased physical activity levels previously reported for this cohort by Wong et al. (2016). For girls, stunting and starting ART later were associated with poor performance on the 6MWT. Jesson et al. (2019) in their multiregional analysis of growth in CLHIV demonstrated that girls had a higher prevalence of stunting than boys at 10 years of age, but showed better subsequent growth that results in boys having a higher prevalence of stunting by 15 years of age (Jesson et al. 2019). It is therefore important to continue to monitor the growth and endurance levels of all children as they go through adolescence as the impairments experienced by children may change. High levels of MVPA and aerobic fitness may also have additional benefits in CLHIV, such as the prevention of cardiovascular risk factors commonly experienced (De Lima et al. 2019) or improving inflammatory profiles (Gopalan et al. 2020).

The fact that both groups had relatively poor levels of endurance in comparison to international studies (Cacau et al. 2016) warrants further investigation. Interestingly the children in the study conducted by Githinji et al. (2017) in Cape Town, South Africa, also walked below average distances on the 6MWT when compared to norms from Switzerland. The fact that a 10-m walkway was used may have contributed to this finding (‘ATS Statement’ 2002). A systematic review that investigated norms for children on the 6MWT found wide variation in normative values for children around the world, thus the authors caution against using norms from a different country as a comparison group (Cacau et al. 2016). Unfortunately, no normal values for the 6MWT exist for South African children, and this is an area that further research can address.

Some of the children in the comparison group may have been exposed to HIV in utero but were uninfected (HEU). The long-term health and development of children who are HEU is a contentious issue with many studies reporting immunological and growth impairment in this group of children (Afran et al. 2014; Jumare et al. 2019). The inclusion of an unknown number of HEU in the comparison group may have biased the results of this group and could explain the lower than expected 6MWT results. Few of the studies discussed have mentioned whether their comparison groups included HEU children or were strictly HIV-unexposed, uninfected children.

## Conclusion

The results of our study suggest that CLHIV who initiated ART within the first 18 months of life and remain on ART have the potential to perform well in assessments of submaximal endurance compared to their uninfected peers. The endurance levels of children, including infected, exposed uninfected as well as unexposed uninfected children, in this community need to be investigated further using more challenging tests, for example, the shuttle run test.

Further studies are needed to evaluate interventions to improve endurance levels and physical activity amongst CLHIV.
